# Removal of the C4-domain preserves the drought tolerance enhanced by CsMYB4a and eliminates the negative impact of this transcription factor on plant growth

**DOI:** 10.1007/s42994-024-00149-5

**Published:** 2024-03-28

**Authors:** Mingzhuo Li, Guoliang Ma, Xiu Li, Lili Guo, Yanzhi Li, Yajun Liu, Wenzhao Wang, Xiaolan Jiang, De-Yu Xie, Liping Gao, Tao Xia

**Affiliations:** 1 State Key Laboratory of Tea Plant Biochemistry and Utilization, Anhui Agricultural University, Hefei, 230036 China; 2 School of Life Science, Anhui Agricultural University, Hefei, 230036 China; 3 Department of Plant and Microbial Biology, North Carolina State University, Raleigh, NC USA; 4 College of Horticulture, Northwest A&F University, Yangling, 712100 China

**Keywords:** Tea, MYB4 repressor, Development, Carbohydrate metabolism, Drought stress, C4-domain

## Abstract

The MYB4 transcription factor family regulates plant traits. However, their overexpression often results in undesirable side effects like growth reduction. We have reported a green tea (*Camellia sinensis*) MYB4 transcription factor (CsMYB4) that represses the phenylpropanoid and shikimate pathways and stunts plant growth and development. In the current study, we observed that in *CsMYB4a* transgenic tobacco (*Nicotiana tabacum*) plants, primary metabolism was altered, including sugar and amino acid metabolism, which demonstrated a pleiotropic regulation by CsMYB4a. The *CsMYB4a* transgenic tobacco plants had improved drought tolerance, which correlated to alterations in carbohydrate metabolism and an increase in proline content, as revealed by metabolic profiling and transcriptomic analysis. To mitigate the undesirable repressive side effects on plant traits, including dwarfism, shrunken leaves, and shorter roots of *CsMYB4a* transgenic plants, we deleted the C4 domain of CsMYB4a to obtain a CsMYB4a-DC4 variant and then overexpressed it in transgenic plants (CsMYB4a-DC4). These CsMYB4a-DC4 plants displayed a normal growth and had improved drought tolerance. Metabolite analysis demonstrated that the contents of carbohydrates and proline were increased in these transgenic plants. Our findings suggest that  an approriate modification of TFs can generate novel crop traits, thus providing potential agricultural benefits and expanding its application to various crops.

**Supplementary Information:**

The online version contains supplementary material available at 10.1007/s42994-024-00149-5.

Dear Editor,

Transcription factors (TFs) play a crucial role in engineering plants for desired traits. Numerous TFs exhibit pleiotropic activities, such as the integration of early maturity, stress tolerance, and high yield (Zhao et al. 2023); OsDREB1C is a typical example of a pleiotropic TF factor that can boost the yield and shorten the growth duration of rice (Wei et al. 2022). In the large repertoire of MYB TFs, numerous MYB4 members have been identified and characterized to repress the plant phenylpropanoid pathway in various ways (Ma and Constabel 2019). An ectopic expression of several *MYB4* transcription factors in tobacco (*Nicotiana tabacum*) has been reported to result in dwarfism, shrunken leaves, white flowers, and shorter roots (Jin et al. 2000; Li et al. 2017; Tamagnone et al. 1998), which arise from the inhibition of lignin, monolignol, and flavonoid biosynthesis. Furthermore, the overexpression of *MYB4* TFs has been reported to increase cell wall degradability and sugar release in various *MYB4* transgenic plants (Fornalé et al. 2010; Jin et al. 2000; Li et al. 2017; Shen et al. 2012; Sonbol et al. 2009; Tamagnone et al. 1998). Meanwhile, the abnormal growth of plants leads to biomass reduction, which is disadvantageous for biofuel production. MYB4 TFs have C1, C2, Zf, and C4 functional domains in the C-terminus. The C2 and C4 motifs have been identified as repressive elements that negatively regulate promoter activity (Shen et al. 2012). In *Arabidopsis thaliana*, the C4 domain of AtMYB4 has been characterized to interact with SAD2/Gir1, an importin β-like protein, facilitating the transport of AtMYB4 into the nucleus (Zhao et al. 2007; Zhou et al. 2015).

We previously reported on a MYB4 transcription factor (*CsMYB4a*) from green tea (*Camellia sinensis*) plants. CsMYB4a was shown to repress the activities of the phenylpropanoid and the shikimate pathways in transgenic tobacco plants (Li et al. 2017), in agreement with earlier reports on *Arabidopsis MYB4* members (Wang et al. 2020). In the current research, we further assessed the potential applications of *CsMYB4a* by examining its effects on transgenic plants. We removed its C4 domain and demonstrated that this modification increased the plant’s drought tolerance and rescued the dwarfism phenotype. Our results indicate that the engineering of TFs can offer new avenues for the development of elite crop traits.

We generated and screened *CsMYB4a* homozygous T2 progeny of transgenic plants. The data showed that the T2 progeny exhibited distinct characteristics, such as dwarfing, shrunken leaves, white flowers, and shorter roots (Li et al. 2017) (Fig. 1A and Fig S1). Other studies have shown that some MYB4 TFs contribute to increased cell wall degradability of and sugar release from transgenic plants (Shen et al. 2012; Sonbol et al. 2009). First, we used GC-MS to analyze various compounds and identified alterations in the carbohydrate metabolism, the TCA cycle, and amino acids in these *CsMYB4a* transgenic plants (Fig. 1B–D). These results showed that, in *CsMYB4a* transgenic tobacco lines 4 and 5, the contents of ribose, fructose, glucose, and sucrose were significantly higher than in wild type plants, with increases ranging from 1.4 to 28-fold (Fig. 1B). Second, the malic acid levels were also increased 3.3 to 4.6-fold in lines 4 and 5 compared to wild type, whereas citric and succinic acids were reduced in their contents. The levels of these acids in line 2 were similar to those of wild type plants or slightly altered (Fig. 1C). Third, the proline levels were 2.1 to 2.4 times higher in CsMYB41 transgenic plants than in wild type plants, whereas the glutamic acid levels were decreased about twofold in lines 4 and 5 (Fig. 1D).

**Fig. 1:**
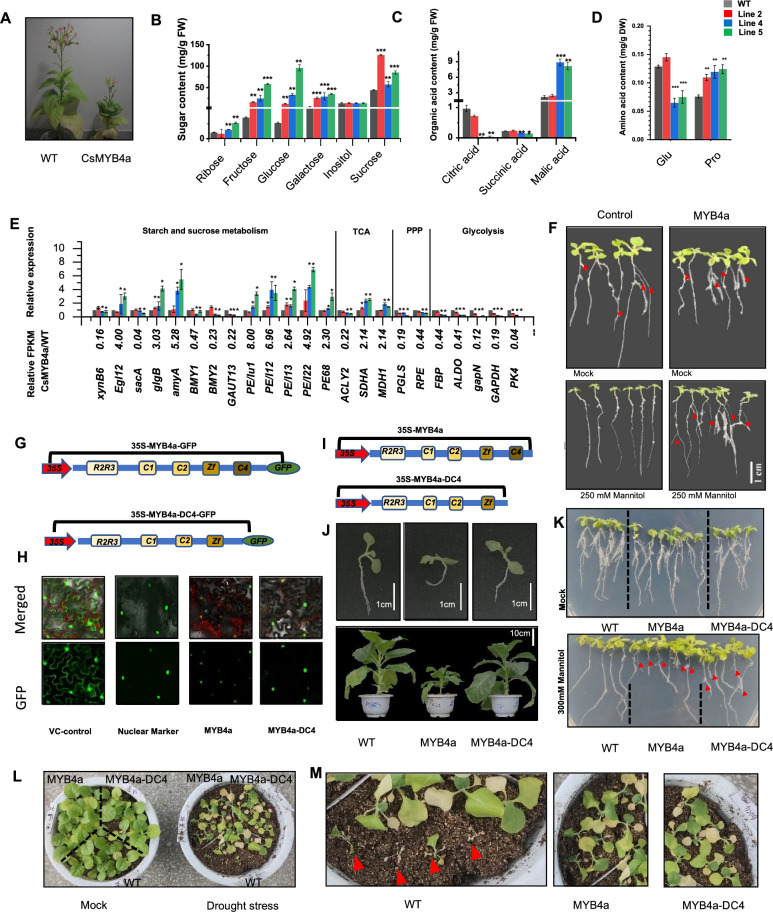
**A**, phenotypes of 50-day-old T2 progeny seedlings of three different *CsMYB4a* transgenic tobacco lines (line2, line4 and line5) versus wild type plants. **B**, Contents of six sugar molecules were compared between wild type and *CsMYB4a* transgenic tobacco plants. **C**, Contents of three organic acids in the TCA-cycle were compared between wild type and *CsMYB4a* transgenic tobacco plants. **D**, Contents of two amino acids, including Glu and Pro were compared between wild type and *CsMYB4a* transgenic tobacco plants. **E**, RPKM values and qRT-PCR data showed transcriptional alterations of 21 genes involved in sugar metabolism. The RPKM value of each gene was obtained from the transcriptomes of *CsMYB4a* transgenic and wild type tobacco plants, which were labeled with RPKMcs and RPKMwt. The ratio of RPKMcs/RPKMwt was calculated to indicate the transcriptional alteration of each gene in these CsMYB4a transgenic plants. RT-qPCR was performed to examine the transcriptional change of each gene. A “*” labeled on each bar indicates the significant difference (*P*-value < 0.05). Green and red arrows are used to visualize that genes from at least two lines are significantly downregulated and upregulated, respectively. *PPP* pentose phosphate pathway, *TCA* tricarboxylic acid cycle. **F**, Phenotypic comparison of 10-day-old WT and *CsMYB4a* transgenic plant grown on MS medium under a 250 mM mannitol stress treatment. Red arrows indicate lateral roots. **G**, Two cloning cassettes were designed for subcellar localization analysis of CsMYB4a and a C4-domain deleted CsMYB4a (CsMYB4a-DC4) **H**, GFP-signal showed that both CsMYB4a and CsMYB4a-DC4 were localized in nuclei in tobacco cells. **I**, Two cloning cassettes were designed for ectopic expression of *CsMYB4a* and *CsMYB4a-DC4* in tobacco. **J**, Growth comparison between wild type *CsMYB4a* and *CsMYB4a-DC4* T2 transgenic 20-day-old and 60-day-old plants. **K**, Phenotype comparison of 15-day-old wild type (WT), *CsMYB4a* and *CsMYB4a-DC4* transgenic plant grown on MS-medium under a 300 mM mannitol stress treatment. Red arrows indicate lateral roots. **L**, Phenotype comparison of 30-day-old WT, *CsMYB4a* and *CsMYB4a-DC4* transgenic plant grown under a drought stress treatment. **M**, Phenotype comparison of WT, *CsMYB4a* and *CsMYB4a-DC4* transgenic plant grown under a drought stress treatment. Red arrows indicate dead plants

Besides metabolic profiling, a previous transcriptome analysis (Li et al. 2017) of *CsMYB4a* transgenic plants revealed 23 differentially expressed genes (DEGs) linked to carbohydrate metabolism, the TCA cycle, and amino acid synthesis (Fig. 1E, Fig.S2, Fig.S3). Specifically, the expression levels of 13 genes were reduced, including five involved in glycolysis, namely *FBP* (fructose-1,6-bisphosphatase), *ALDO* (fructose-bisphosphate aldolase), *GAPDH* (glyceraldehyde-3-phosphate dehydrogenase), gapN (glyceraldehyde-3-phosphate dehydrogenase) and *PK4* (pyruvate kinase); two in the pentose phosphate pathway, namely *PGLS* (6-phosphogluconolactonase) and *RPE*(ribulose-phosphate 3-epimerase); five in starch and sucrose metabolism, namely *xynB6* (xylan 1,4-beta-xylosidase), *sacA* (alpha-1,4-galacturonosyltransferase), *BMY1* (beta-amylase1), *BMY2* (beta-amylase2), and *GAUT* (alpha-1,4-galacturonosyltransferase); and one in the TCA cycle, ACLY2 (ATP citrate lyase2). By contrast, the expression levels of 10 genes were increased, with eight involved in starch and sucrose metabolism and two (SDHA, succinate dehydrogenase complex assembly factor, MDH1, malate dehydrogenase) associated with the TCA cycle. These transcriptional changes were confirmed by qRT-PCR (Fig. 1E).

We analyzed promoter sequences (1.6 kb long nucleotide upstream of coding sequence region) of the abovementioned genes to identify AC-elements (type I, type II, type III and type IV) (Fig. S4 A) to which CsMYB4a can bind. This analysis identified four types of AC elements from 10 promoters including *gapN* of glycolysis, *PGLS* and *RPE* of PPP, *ACLY2* and *MDH1* of TCA, and *BMY1*, *PE/I12* (pectinesterase12), and *PE/I13* (pectinesterase13) of starch and sucrose metabolism. CsMYB4a was shown to moderately down-regulate the promoter activity of several genes, including *NtGAPN*, *NtPGLS*, *NtACLY2* and *NtBMY1*. Dual-luciferase assays showed CsMYB4a reduced the promoter activity by 22% to 37% for the selected genes. These results suggest that it affects sugar metabolism by modulating gene expression (Fig. S4 B).

Given that sugar metabolism, under different osmotic conditions, can affect plant development, we assessed the development of wild type and *CsMYB4a* tobacco seedlings growing on MS medium supplemented with different mannitol concentrations from 0 and 250 mM. The resulting data revealed that those *CsMYB4a* transgenic tobacco seedlings increased their tolerance to drought, as shown by their ability to develop normal lateral roots under a 250 mM mannitol treatment, a condition under which wild type seedlings could barely survive (Fig. 1F and Fig. S5 A, B). These data suggest that the *CsMYB4a* transgene improves the plant resilience to drought stress.

To explore the mechanisms underlying dwarfism in these *CsMYB4a*-overexpressing plants, we tesed its C4 domain.  Based on reports that the C4-domain of AtMYB4 is associated with nuclear localization and repression of the phenylpropanoid pathway (Zhao et al. 2007; Zhou et al. 2015), we hypothesized that manipulation of the C-4 domain would rescue the dwarfism phenotype of *CsMYB4a* overexpressing tobacco plants. A deletion of the nucleotide sequences encoding the C4 domain of *CsMYB4a* (resulting in *CsMYB4a-DC4*) was completed to test this hypothesis (Li et al. 2017). We observed that despite the absence of the C4-domain, CsMYB4a-DC4 was still localized in the nuclei. This result was in contrast with the reports of AtMYB4, which was not localized in the nuclei after the deletion of its C4 domain (Fig. 1G and H). This result indicates that the C4 domain of CsMYB4a is not necessary for its nuclear localization (Zhao et al. 2007; Zhou et al. 2015).

A binary construct, with *CsMYB4a-DC4* driven by the *35S*-promoter, was transformed into tobacco plants (Fig. 1I). These *CsMYB4a-DC4* transgenic plants exhibited growth comparable to wild type (Fig. 1J), revealing the C-4 domain's involvement in the growth inhibition. Next, T2 progeny of *CsMYB4a-DC4* plants, *CsMYB4a*, and wild type plants were germinated on MS medium containing 0 or 300 mM mannitol. *CsMYB4a-DC4* and *CsMYB4a* transgenic seedlings developed more lateral roots than wild type under this 300 mM mannitol condition, suggesting enhanced drought tolerance (Fig. 1 K, Fig S5 C, D). Finally, to test the tolerance of these plants to draught stresses, seedlings of *CsMYB4a* and *CsMYB4a-DC4* transgenic plants and wild type plants were grown, side by side, for three weeks under the drought treatments. Under the same drought condition, the survival rate of wild type plants was lower than 60%, whereas that of all *CsMYB4a* and *CsMYB4a-DC4* transgenic plants was 100% (Fig. 1L and M, Fig S8). Furthermore, we analysed amino acids and sugar in 5-week-old seedlings of wild type and *CsMYB4a-DC4* transgenic plants. These assays diclosed revealed that, in the *CsMYB4a-DC* plants, the glutamate and proline contents were increased by 10% and 30%, respectively (Fig S6); the contents of fructose, glucose/galactose, and sucrose were increased by 26%, 23%, and 37%, respectively (Fig S7). These data suggested that the increased Pro and sugar contents might be associated with the tolerant enhancement of the transgenic plants to drought stress.

A modification of TFs may be an appropriate strategy to develop elite plant traits. TFs, especially the MYB TFs, are useful in engineering plants (Ambawat et al. 2013). Overexpressing *MYB4* TFs in plants can result in dwarfism and other abnormalities, by inhibiting shikimate and phenylpropanoid metabolism (Li et al. 2017). Also, the overexpression of MYB4 TFs was shown to increase cell wall degradability and sugar release (Sonbol et al. 2009), which is beneficial for biofuel engineering. However, the repressive effects of MYB4 TFs on plant growth can reduce biomass, a disaventage for for biofuel production. MYB4 TFs are characterized by possessing multiple functional domains, including C1 (unclear), C2 (repressive), Zf (unclear) and C4 (repressive, nuclear localizing) (Shen et al. 2012). Based on these features, we removed the C4 domain of CsMYB4a and obtained a truncated variant CsMYB4a-DC4, which was still localized in the nucleus in tobacco plants. The transgenic plants expressing this variant grew normally and retained the drought resistance feature, suggesting that removing the C4 domain was a beneficial strategy.

We observed that the proline and sugar contents in *CsMYB4a-DC4* transgenic plants were increased by 10% to 37%. This change was associated with the plants’ tolerance to drought stress. The ectopic expression of *CsMYB4a-DC4* led to metabolic alterations, albeit to a lesser extent than the full-length *CsMYB4a*. These changes were still effective in enhancing drought resilience. Altough the impact of *CsMYB4a-DC4* on gene expression level and promoter activity changes that are associated with metabolisms in *CsMYB4a-DC4* plants remains to be explored, our findings indicate the roles of domains in specific plant growth.

Taken together, the *CsMYB4a* overexpression hinders growth but significantly improves the tolerance to osmotic and drought stresses. The removal of the repressive C-4 domain of *CsMYB4a* not only eliminates the growth abnormalities but also boosts stress resilience. Metabolic and transcriptomic analyses suggest that these stress tolerances are related to the changes of carbohydrate metabolism and the increase of proline. These findings show the potential of transcription factors in engineering to introduce beneficial traits into crops.

## Materials and methods

### Genotypes, selection of T2 progeny, and plant growth

Three *CsMYB4a* transgenic lines 3, 4, and 5 reported previously (Li et al. 2017) were used in this study. The selection of T2 progeny followed the method previously described. The T2 progeny of line 3, 4 and 5 were confirmed using both genomic DNA based PCR and RT-PCR. Wild type *Nicotiana tabacum* cv. “G28” genotype and T2 progeny of lines 3, 4, and 5 grown in the greenhouse as described previously (Li et al. 2017).

### Deletion of C-4 domain, development of constructs, confocal microscopy analysis, and genetic transformation

The *CsMYB4a-DC4* was obtained directly by PCR and the primers were listed in supplemental Table S1. The CsMYB4a-DC4 was cloned into the plasmid PCB2004 and PGWB5 for transgenic and subcellar localization analysis. The plasmid construction method was followed as described previously (Li et al. 2017). The plant transformation and confocal microscopy analysis were also followed by the same method that we described before (Li et al. 2017).

### Drought stress treatments

Wild type, *CsMYB4a*, and *CsMYB4a-DC4* transgenic plant seeds were sterilized and germinated on MS medium (30 g/L sucrose, 4.74 g/L Murashige and Skoog Basal Medium (Sigma, USA), 8 g/L agar, 0.01 g/L VC, pH = 5.8), under controlled conditions (temperature 25 humidity 60%). After 7 days, similarly sized seedlings were transferred to MS media with varying mannitol concentrations mannitol (0, 50, 150, 250 and 300 mM) to assess drought tolerance. Additionally, to directly test drought stress tolerance, seedlings were planted in soil, with three replicates of 20 plants each per genotype. The experiment included a drought group, which wasn't watered, and a control group, watered daily for 21 days, after which survival rates were calculated.

### Data mining of transcriptome

Transcriptomes were developed from two genotypes, wild type, and line 4 plants which has been described before (Li et al. 2017). cDNA libraries were constructed according to a previously described method (Tai et al. 2015) and sequencing was conducted using the Illumina HiSeq^™^ 2000 platform. The resulting reads, RPKM values analysis, data mining and the identification of unigenes, biosynthetic pathway analyses were completed following the previous methods described already.

### Quantitative PCR (qRT-PCR) analysis

The qRT-PCR analysis was performed to analyze expression levels of 47 genes in T2 progeny of three lines and wild-type tobacco plants using SYBR-Green PCR Mastermix (Invitrogen, Carlsbad, CA) on a CFX96^™^ (Bio-RAD, California, USA). Forty-seven gene-specific primer pairs and their respective thermal programs used are listed in Supplementary Table S1.

### Identification of AC-elements and promoter cloning, and dual luciferase assay

Twenty-three promoter sequences of genes involved in glycolysis, PPP, TCA, and sugar metabolism were identified from the genomic sequences of tobacco (http://www.ncbi.nlm.nih.gov/). Each sequence consisted of 1.6 kb of nucleotides located at the immediate upstream of the coding region. AC-elements were identified using the Plantpan approach (Chow et al. 2019). At least 1 kb of nucleotides at the immediate upstream of the coding regions of *NtGAPN*, *NtPGLS*, *NtACLY2* and *NtBMY1* from tobacco plants were cloned into the PMD19-T plasmid included in the Genome-walker kit (Takara, Dalian, China) and then sequenced with the service at BGI. A dual luciferase assay was conducted to analyze the regulatory activity of *CsMYB4a* on the above promoters. The coding region of *CsMYB4a* was inserted to the Gateway overexpression vector PCB2004 to obtain a new effector vector, PCB2004-CSMYB4a, as reported previously (Li et al. 2017). PCB2004 was used as an effector control. The dual luciferase experiments protocol was followed the previous report by CsMYB4a to phenylpropanoids gene promoters (Li et al. 2017).

### Extraction and derivatization of sugars and organic acids

Metabolite extraction was performed, as previously reported (Zhang et al. 2011). A 0.15 g tobacco leaf sample was extracted, twice, using 2 mL of methanol:chloroform:water (2.5:1:1) for 30 min and centrifuged at 5000 g for 10 min, and then pooled. The residue was then extracted with 1 mL of methanol:chloroform (1:1) under the same conditions, and the supernatant was combined with the previous ones. The volume was adjusted to 5 mL with methanol:chloroform (1:1), and 0.5 mL deionized water was added and mixed before centrifugation at 5000 g for 5 min. The upper water–methanol phase was collected, and then the volume was adjusted to 5 mL with 50% methanol. Ribitol (0.2 mg/mL) was added as an internal standard. 100 µL of the water-methanol extract was dried and the residue was dissolved in 100 µL of methyl amine hydrochloride (20 mg/mL in pyridine). The resulting mixture was incubated at room temperature for 50 min, followed by silylation with 60 µL of TSTFA at 70 °C for 2 h. Each sample was adjusted to 260 µL for GC–MS analysis.

### GC–MS analysis

Derivatives of organic acids and sugars were analyzed using gas chromatography-mass spectrometry (GC–MS) analysis on a SHIMADZU QP2010 (Shimadzu Instruments, Kyoto, Japan) coupled to a Fisons MD800 quadrupole mass detector (Fisons Instruments, CA, USA). 1 µl of sample was injected into a split injector (1:10). Metabolites were separated in a fused-silica capillary column (0.25 lm DB-5 MS stationary phase, J and W Scientific, Folsom, CA, USA) with a constant pressure mode set at 91 kPa and an injection temperature of 250 ºC. A gradient oven temperature program for GC was developed to separate the metabolites as described in a previous report (Zhang et al. 2011).

## Supplementary Information

Below is the link to the electronic supplementary material.

Supplementary file1 (PDF 525 KB)

## Data Availability

The data are available from the corresponding author upon reasonable request.
