# Publisher Correction: Removal of the C4-domain preserves the drought tolerance enhanced by CsMYB4a and eliminates the negative impact of this transcription factor on plant growth

**DOI:** 10.1007/s42994-024-00163-7

**Published:** 2024-05-06

**Authors:** Mingzhuo Li, Guoliang Ma, Xiu Li, Lili Guo, Yanzhi Li, Yajun Liu, Wenzhao Wang, Xiaolan Jiang, De-Yu Xie, Liping Gao, Tao Xia

**Affiliations:** 1 State Key Laboratory of Tea Plant Biochemistry and Utilization, Anhui Agricultural University, Hefei, 230036 China; 2 School of Life Science, Anhui Agricultural University, Hefei, 230036 China; 3 Department of Plant and Microbial Biology, North Carolina State University, Raleigh, NC USA; 4 College of Horticulture, Northwest A&F University, Yangling, 712100 China

**Publisher Correction: aBIOTECH** 10.1007/s42994-024-00149-5

The original article has been updated to correct two errors introduced during production:

In this article Xiu Li the same as Mingzhuo Li and Guoliang Ma should have been denoted as equally contributing authors.

Furthermore, Fig. 1 was revised for minor formatting corrections. Revised Fig. 1 is as follows:

**Fig. 1:**
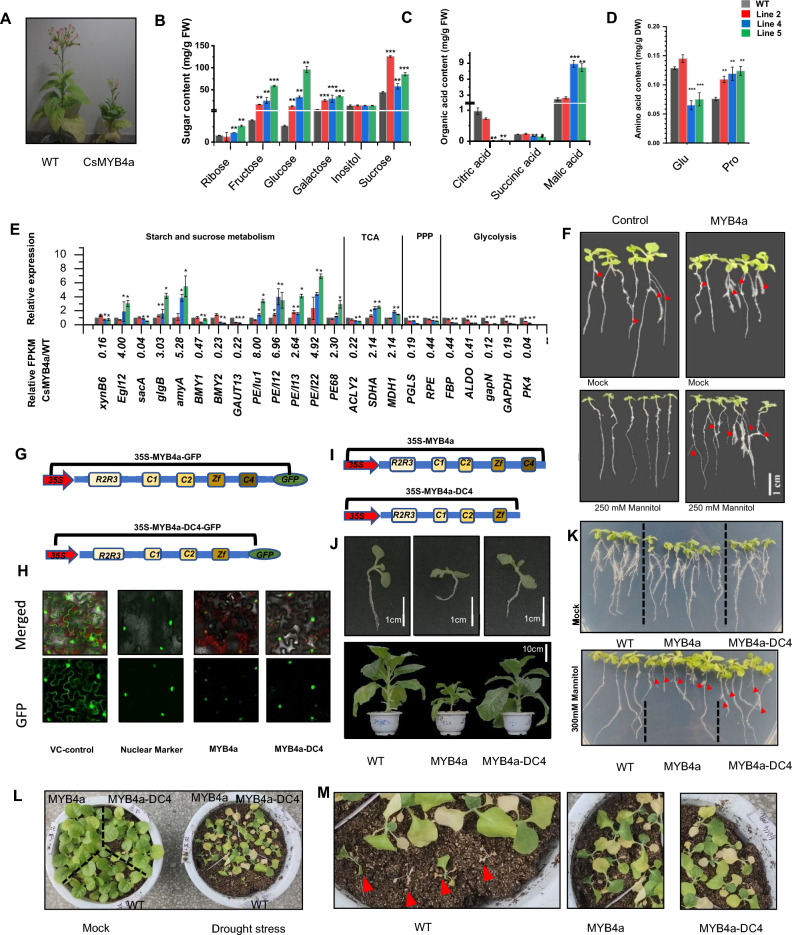
**A** Phenotypes of 50-day-old T2 progeny seedlings of three different CsMYB4a transgenic tobacco lines (line2, line4 and line5) versus wild type plants. **B** Contents of six sugar molecules were compared between wild type and CsMYB4a transgenic tobacco plants. **C** Contents of three organic acids in the TCA-cycle were compared between wild type and CsMYB4a transgenic tobacco plants. **D** Contents of two amino acids, including Glu and Pro were compared between wild type and CsMYB4a transgenic tobacco plants. **E** RPKM values and qRT-PCR data showed transcriptional alterations of 21 genes involved in sugar metabolism. The RPKM value of each gene was obtained from the transcriptomes of CsMYB4a transgenic and wild type tobacco plants, which were labeled with RPKMcs and RPKMwt. The ratio of RPKMcs/RPKMwt was calculated to indicate the transcriptional alteration of each gene in these CsMYB4a transgenic plants. RT-qPCR was performed to examine the transcriptional change of each gene. A ‘‘*’’ labeled on each bar indicates the significant difference (P value < 0.05). Green and red arrows are used to visualize that genes from at least two lines are significantly downregulated and upregulated, respectively. PPP pentose phosphate pathway, TCA tricarboxylic acid cycle. **F** Phenotypic comparison of 10-day old WT and CsMYB4a transgenic plant grown on MS medium under a 250 mM mannitol stress treatment. Red arrows indicate lateral roots. **G** Two cloning cassettes were designed for subcellar localization analysis of CsMYB4a and a C4-domain deleted CsMYB4a (CsMYB4a-DC4).** H** GFP-signal showed that both Cs- MYB4a and CsMYB4a-DC4 were localized in nuclei in tobacco cells. **I** Two cloning cassettes were designed for ectopic expression of CsMYB4a and CsMYB4a-DC4 in tobacco. **J** Growth comparison between wild type CsMYB4a and CsMYB4a-DC4 T2 transgenic 20-day-old and 60-day-old plants. **K** Phenotype comparison of 15-day-old wild type (WT), CsMYB4a and CsMYB4a-DC4 transgenic plant grown on MS-medium under a 300 mM mannitol stress treatment. Red arrows indicate lateral roots. **L** Phenotype comparison of 30-day-old WT, CsMYB4a and CsMYB4a-DC4 transgenic plant grown under a drought stress treatment. **M** Phenotype comparison of WT, CsMYB4a and CsMYB4a-DC4 transgenic plant grown under a drought stress treatment. Red arrows indicate dead plants

