# Supplementary material for: Removal of the C4-domain preserves the drought tolerance enhanced by CsMYB4a and eliminates the negative impact of this transcription factor on plant growth
Source: aBIOTECH. 2024 Mar 28;5(3):368–74. doi: 10.1007/s42994-024-00149-5 (PMC11399494; doi:10.1007/s42994-024-00149-5)
Supplement: Supplementary file 1 — Supplementary file1 (PDF 525 KB) [file 42994_2024_149_MOESM1_ESM.pdf]

Supplementary Information

Table of contents

Fig S1: Phenotypic comparison of leaf (A-B), flower (C), and root (D) between T2 CsMYB4a transgenic tobacco (line5) and wild-type plants.

Fig S2: Effects of the CsMYB4a overexpression on transcriptional levels of carbohydrate metabolism genes.

Fig S3: Unigene groups resulted from functional annotation of transcriptome revealing genes involved in sugar, PPP and TCA metabolisms in CsMYB4a transgenic tobacco plants.

Fig S4: AC-elements localizations in the promoter of eight genes involved in carbohydrate metabolisms in tobacco plants.

Fig S5: The quantitative statistics result of root length and lateral root number of WT, *CsMYB4a*, and *CsMYB4a-DC4* transgenic plant under the mannitol stresses.

Fig S6: The amino acids concentration in WT and *CsMYB4a-DC4* transgenic tobacco plants.

Fig S7: The carbohydrate metabolites concentration in WT and *CsMYB4a-DC4* transgenic tobacco plants.

Fig S8: The survival rate of WT, *CsMYB4a*, and *CsMYB4a-DC4* transgenic young seedlings under three weeks of drought stress.

Table S1: Oligonucleotide primers were used in the experiments.

Fig S1

A B C D

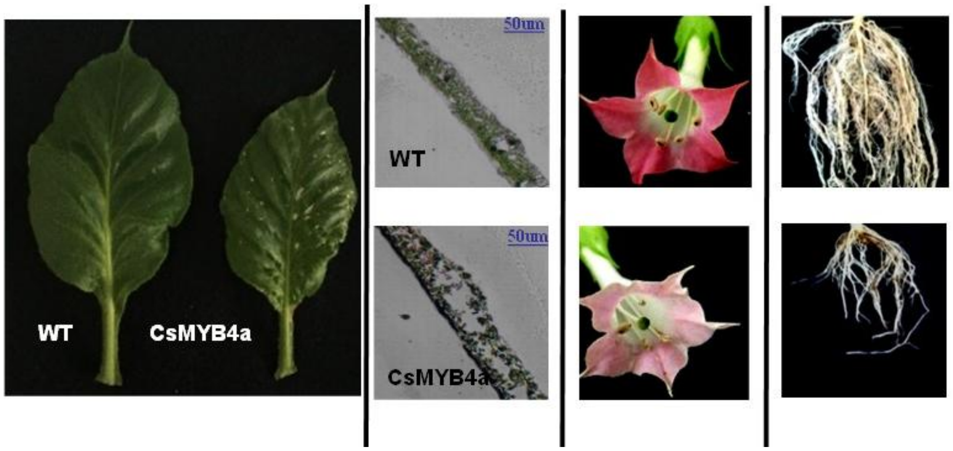

Fig S1: Phenotypic comparison of leaf (A-B), flower (C), and root (D) between T2 CsMYB4a transgenic tobacco (line5) and wild-type plants.

Fig S2

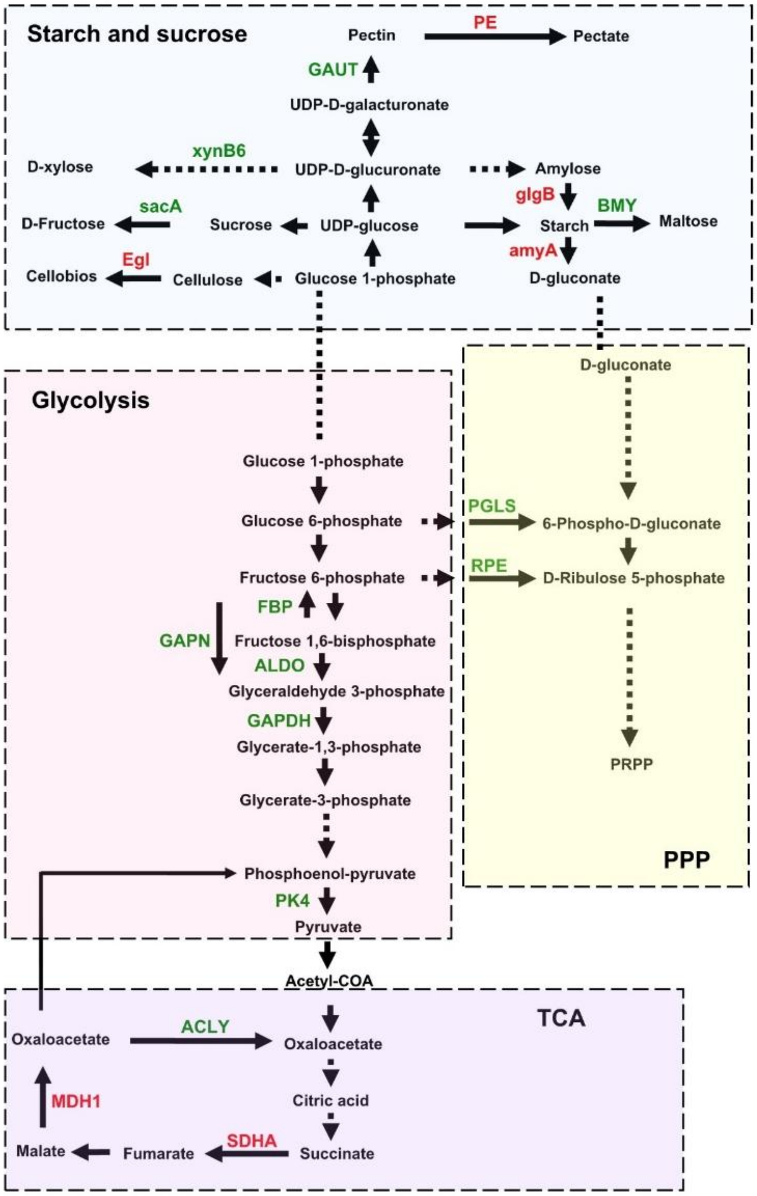

Fig S2: Effects of the CsMYB4a overexpression on transcriptional levels of carbohydrate metabolism genes. Steps with enzyme names show the sugar metabolic pathway and tricarboxylic acid (TCA) cycle. Next-generation sequencing was carried out and the RPKM value of each gene was calculated to compare their expression levels in transgenic vs. wild-type plants. Enzymes highlighted with red or green color are upregulated or downregulated in RPKM values of their transcripts in leaves of CsMYB4a transgenic plants. The green color indicates down-regulated genes, while the red color indicates up-regulated genes. PPP: pentose phosphate pathway; PRPP: Phosphoribosyl pyrophosphate.

Fig S3

A **B**

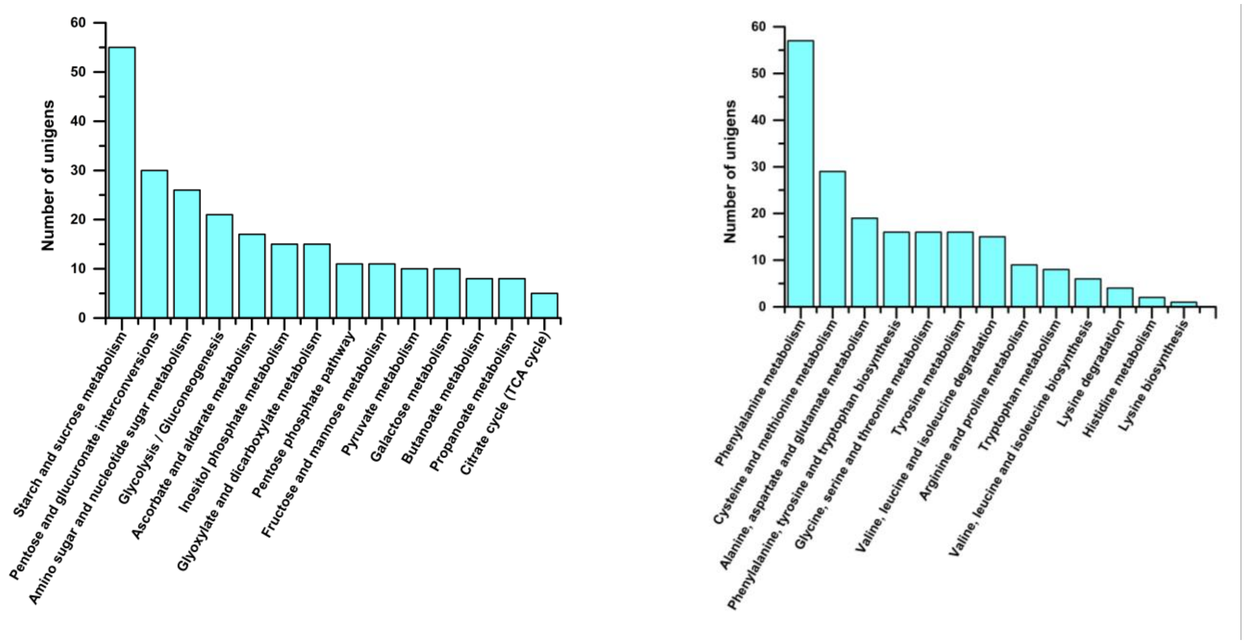

Fig S3: Unigene groups resulted from functional annotation of transcriptome revealing genes involved in sugar, PPP and TCA metabolisms in CsMYB4a transgenic tobacco plants. Unigenes from the transcriptomes were metabolically annotated to metabolisms of sugar, PPP, and TCA. PPP: pentose phosphate pathway; TCA: tricarboxylic acid cycle.

Fig S4

A

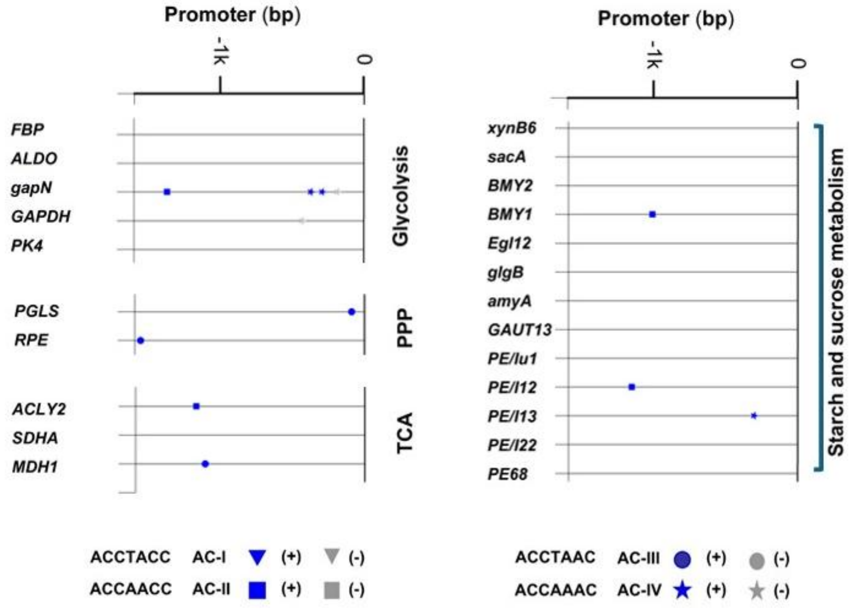

B

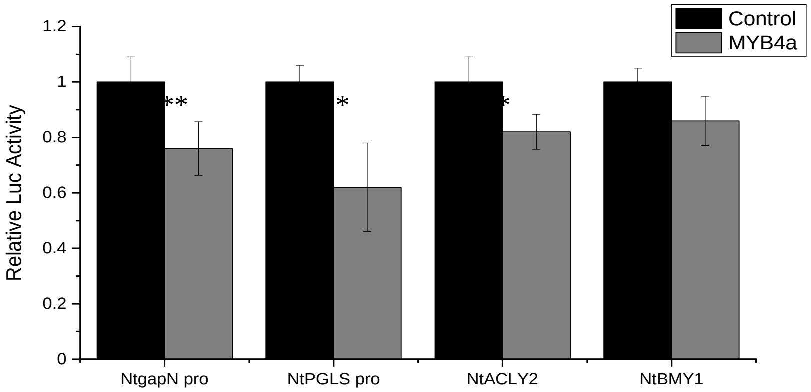

Fig S4: AC-elements localizations in the promoter of eight genes involved in carbohydrate metabolisms in tobacco plants. A, the locations of four AC-element types in the promoter regions genes with an altered expression, in the starch and sucrose metabolism, glycolysis pathway, PPP pathway, and TCA cycle in CsMYB4a transgenic tobacco plants. B, the dual luciferase (LUC) assays show that CsMYB4a reduces the promoting activity of NtGAPN, NtPGLS, NtACLY2 and NtBMY1 promoters. CsMYB4a was used as the effector and the two promoters fused with the firefly luciferase gene were used as the reporter. Empty vector was used as the effector as the control. The promoters of NtGAPN and NtPGLS were fused with luciferase. (*n*=3 experiments, *$P<0.05$, **$P<0.01$). Con: control vector.

Fig S5

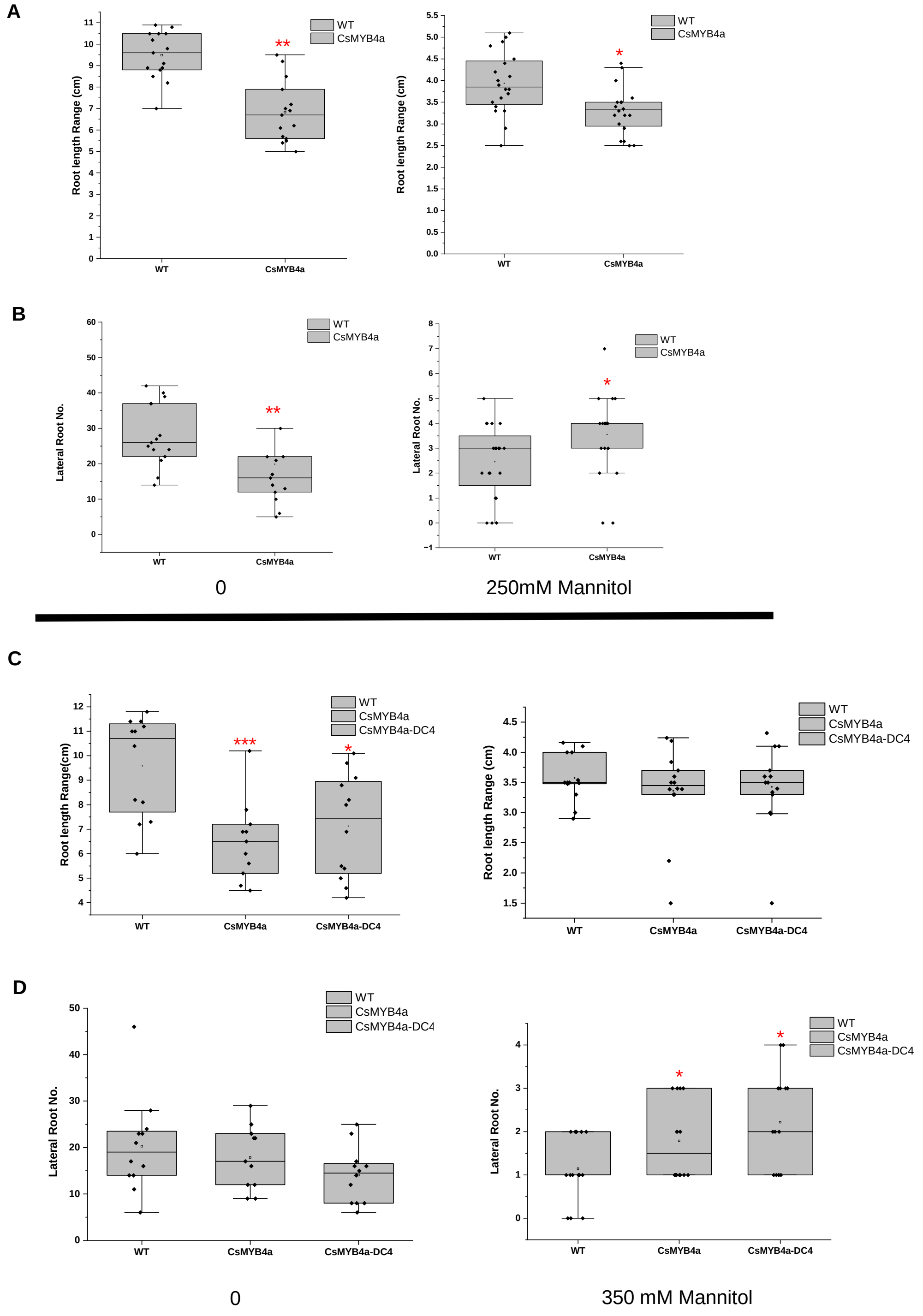

Fig S5: The quantitative statistics result of root length and lateral root number of WT, *CsMYB4a*, and *CsMYB4a-DC4* transgenic plant under the mannitol stresses. A, The root length quantitative statistics data

of the WT and *CsMYB4a* transgenic tobacco seedlings under 0 and 250mM mannitol stress. B, The lateral root number quantitative statistics data of the WT and *CsMYB4a* transgenic tobacco seedlings under 0 and 250mM mannitol stress. C, The root length quantitative statistics data of the WT, *CsMYB4a*, and *CsMYB4a-DC4* transgenic tobacco seedlings under 0 and 350mM mannitol stress. D, The lateral root number quantitative statistics data of the WT, *CsMYB4a*, and *CsMYB4a-DC4* transgenic tobacco seedlings under 0 and 350mM mannitol stress. (n = 3, $*P < 0.05$, $**P < 0.01$, $***P < 0.001$)

Fig S6

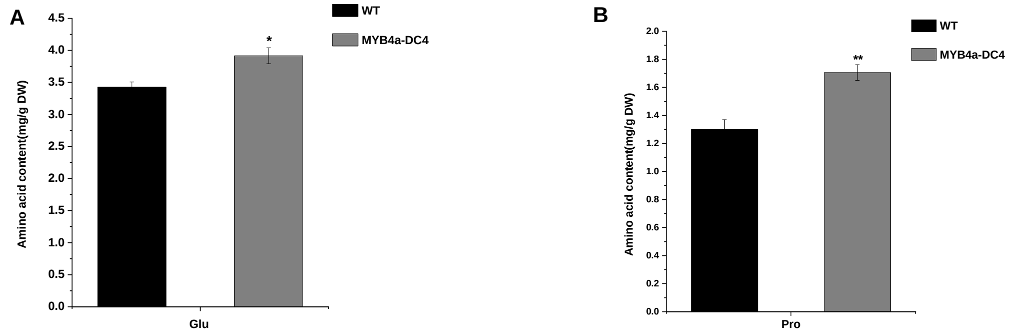

Fig S6. The amino acids concentration in WT and *CsMYB4a-DC4* transgenic tobacco plants. A, the glutamic acid concentration in WT and CsMYB4a-DC4 transgenic tobacco plants. B, the proline concentration in WT and CsMYB4a-DC4 transgenic tobacco plants. (n = 3, $*P < 0.05$, $**P < 0.01$, $***P < 0.001$)

Fig S7

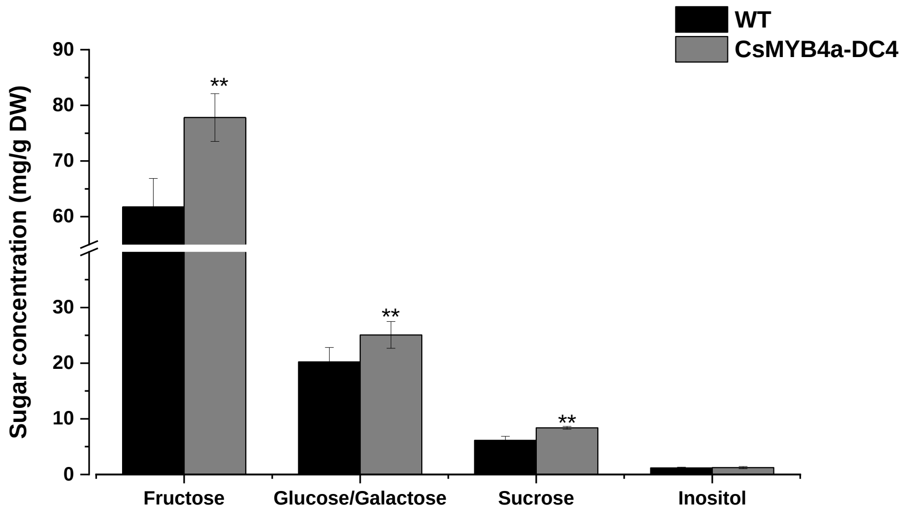

Fig S7. The carbohydrate metabolites concentration in WT and *CsMYB4a-DC4* transgenic tobacco plants. (n = 3, $*P < 0.05$, $**P < 0.01$, $***P < 0.001$)

Fig S8

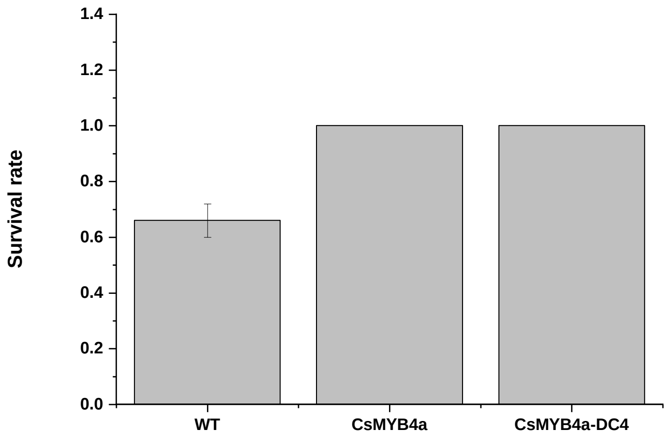

Fig S8. The survival rate of WT, *CsMYB4a*, and *CsMYB4a-DC4* transgenic young seedlings under three weeks of drought stress.

Supplementary Table 1. Oligonucleotide primers were used in the experiments.

	Primers	Sequence
**qRT-PCR in tobaccos**	Actain- F	TAGAAACCCCAAGTACCCTCG
	Actain- R	TGCTTTCTTCGTCCCATCAG
	PK4-F	AAATGGAAAGGACGATGCTGAA
	PK4-R	CACCACATGGCAACAAGGAAA
	GAPDH-F	CTGACAAGAAGATCAAGATCGGAA
	GAPDH-R	GAGATAAATGGGTCGTTCACTGC
	gapN-F	CATGTACACAAGAAGAGGTGAACAAG
	gapN-R	ACAACCTCAGTGACAGCATCTTTAG
	ALDO-F	CATCTTTGGAAGGCACTCTGTAA
	ALDO-R	CATTCATCACGAAGTACAAGCACA
	FBP-F	TCCTGAATCCCGTGGTGACTT
	FBP-R	CTCCACAAATGTAGCCTCTTCG
	RPE-F	CTGCGACTGGATTCATGTAGATGT
	RPE-R	CAAGTGGATGGTAGAAGATTGCTC
	PGLS-F	TGCGGCAGATGATTACGAGA
	PGLS-R	TGGCGGATGAGTTTATTACAGG
	MDH1-F	GATCTCAGGTTTCTGGGTTTGC
	MDH1-R	CAGTGGAGTTCACAGGGTTGC
	SDHA-F	AATCCAAGCAGCAGCCAAAC
	SDHA-R	GGACCCACAGATCCCTTCTCTAC
	ACLY2-F	AGCCTGTTGTTGCCTGGGT
	ACLY2-F	AATGCTTCTTTGATTGCTCCTTC
	PE68-F	GCAGAGTGTAGCAGTGTATCAGCA
	PE68-R	AGAAGAGGATGGGCAGGAGG
	PE/I22-F	TGTGGGAGATGGGATTGGAG
	PE/I22-F	AGAGAATGGGCGTAAAGTGTGTC
	PE/I13-F	ATGTGTGTGGACGGATACCCTG
	PE/I13-R	GCAATACCCTTTGGATCTTTACCT
	PE/I12-F	ATGGGCTCCTCCTTTATTACGAA

	PE/I12-R	CCTGTTCCAGATAAGGCTGATTG
	PE/Iu1-F	ACCGATACAGACATTGCTAATGAGA
	PE/Iu1-R	TTCGGAACATCTACATTCTCCCT
	GAUT13-F	AGTTGTCTACTTGGATTCCGATTTG
	GAUT13-R	TCGATTACCATTACTCCCGTGTT
	BMY2-F	AGTGCTGGTGGGGTATAGTGGA
	BMY2-R	CATCGTGGAAGAGGAATATGAACA
	BMY1-F	GCAACTGGTATGTTGGCTTCC
	BMY1-R	AACTGTCGTGATGGCTATTCTCC
	amyA-F	AGGACGGAATTTCTACTTTGGTG
	amyA-R	TGCTCCCATACAACATAGTCCTG
	glgB-F	TTTTCCCAGATGCGATTACCA
	glgB-R	CACTCTCCAATCCTCATCCCTT
	scaA-F	CTTTGACCCTAGCAAGAACCG
	scaA-R	TGTCCAAACTTGCGAACGAG
	Egl12-F	CACCAGTAATCCCAATCCTCATA
	Egl12-R	AGTGTTGGCTCTGTATAGCTGAAA
	xynB6-F	GTTATCCTGGAAGAACCTATCGG
	xynB6-R	AACCAAGCCTACTGCGTCCT
**Primers for dual-luciferase essays for tobacco promoters**	GAPN Pro-F	GGGGACAAGTTTGTACAAAAAAGCAGGCT TTTCAAAGAACAAGCATAAAATAGG
	GAPN Pro-R	GGGGACCACTTTGTACAAGAAAGCTGGGTTTGATTGGTAA AAATGGCAGGTA
	PGLS Pro-F	GGGGACAAGTTTGTACAAAAAAGCAGGCT TTTGTGTTAGAACTTTCATAAG
	PGLS Pro-R	GGGGACCACTTTGTACAAGAAAGCTGGGTTTGTTTATATAG GAATGGAGAGTGAGAAA
	ACLY Pro-F	GGGGACAAGTTTGTACAAAAAAGCAGGCT TTTGAATCTCTCTCGTGTTTCGT
	ACLY Pro-R	GGGGACCACTTTGTACAAGAAAGCTGGGTTTGTCTGCTGAT TTAATCGAGATCTC
	BMY1 Pro-F	GGGGACAAGTTTGTACAAAAAAGCAGGCT TTTGATATGCGTATACACTATCCT

	BMY1 Pro-R	GGGGACCACTTTGTACAAGAAAGCTGGGTTTGAATATTCAGAAAATTTTGAAT
**Primers for CsMYB4a-DC4**	CsMYB4a-F	atgggaaggtcaccttgctgtgagaa
	CsMYB4a-DC4-R	agtgttgctgctactagttcca
